# Administration of Nebulized Nitroglycerin Inhalation Combined With Subarachnoid Anesthesia for Cesarean Delivery With Pulmonary Arterial Hypertension: A Case Report

**DOI:** 10.1002/ccr3.70916

**Published:** 2025-09-29

**Authors:** Lian Xin, Chunxiao Hu, Yiling Qian, Shunmei Lu, Jingjing Xu, Zhengfeng Gu

**Affiliations:** ^1^ Department of Anaesthesiology, The Affiliated Wuxi People's Hospital of Nanjing Medical University, Wuxi People's Hospital, Wuxi Medical Center Nanjing Medical University Wuxi Jiangsu China

**Keywords:** case report, cesarean delivery, nebulized nitroglycerin inhalation, pulmonary arterial hypertension, spinal anesthesia

## Abstract

In patients with severe pulmonary hypertension, nebulized nitroglycerin inhalation combined with subarachnoid anesthesia is a safe anesthetic option for cesarean section. It maintains stable hemodynamics and, by decreasing pulmonary artery pressure, prevents right heart failure.

## 1 Introduction

Cesarean delivery associated with congenital heart disease and severe pulmonary hypertension is rare and has high morbidity and mortality in gravida, even with modern management strategies [1, 2]. The successful use of nebulized nitroglycerin inhalation (NNI) in children and gravida with congenital heart disease to decrease pulmonary arterial hypertension has been reported [3, 4]. However, the use of NNI combined with dexmedetomidine in subarachnoid anesthesia (SA) for cesarean delivery has not been reported. We report a case of severe congenital heart disease and pulmonary arterial hypertension in a 26‐year‐old woman. This case report was prepared using the CARE guidelines [5].

## 2 Case History

**FIGURE 1:**
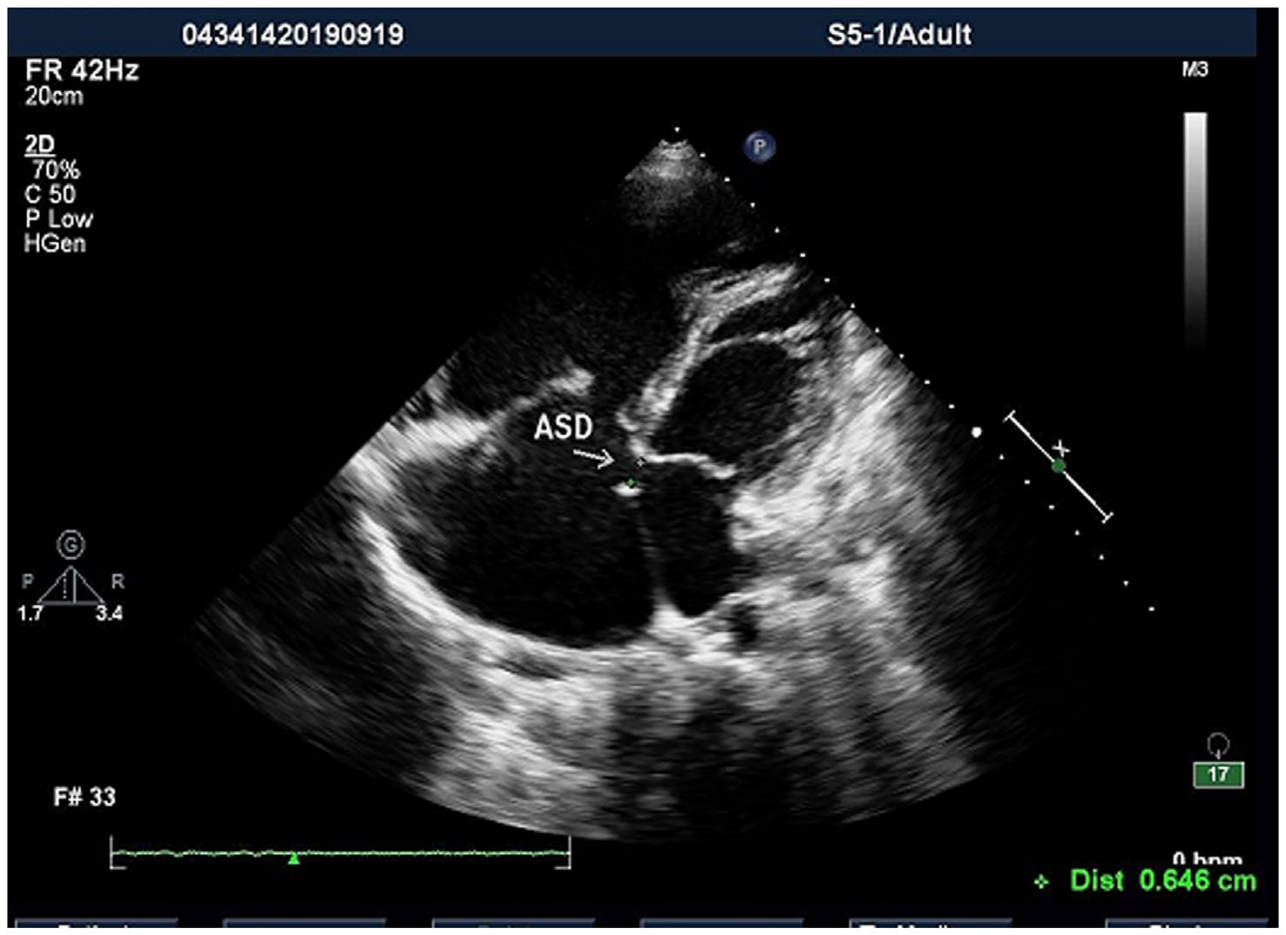
Preoperative atrial septal defect. ASD, atrial septal defect, white arrow.

**FIGURE 2:**
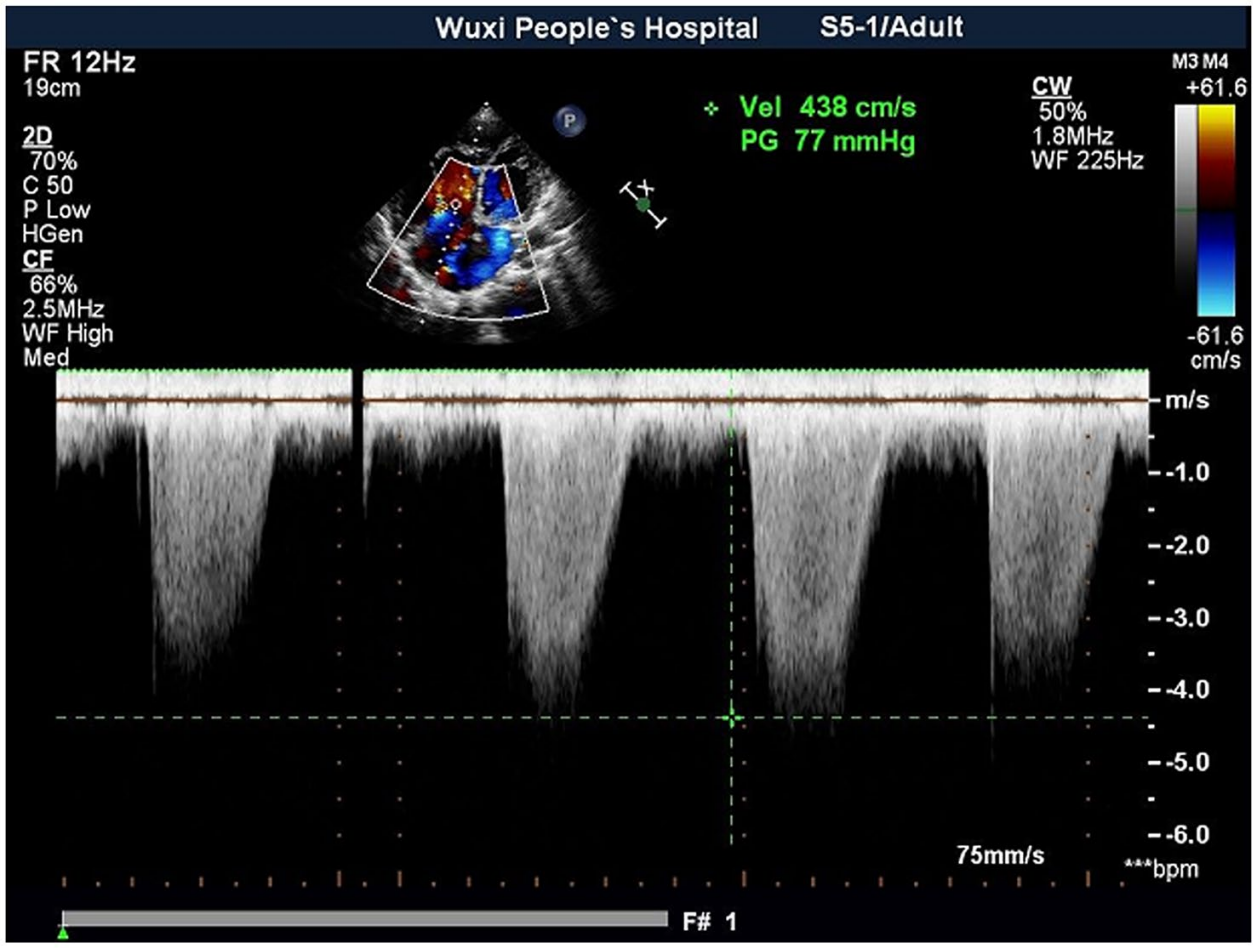
Preoperative pulmonary artery hypertension. PG, pressure gradient.

A 26‐year‐old patient (height: 155 cm, weight: 65 kg) was scheduled to undergo cesarean delivery under general anesthesia with venoarterial extracorporeal membrane oxygenation (VA‐ECMO). She was diagnosed at 32 weeks of gestation with congenital heart disease; a 6‐mm atrial septal defect (Figures 1 and 2); bidirectional shunt; partial anomalous pulmonary venous drainage; pulmonary arteriovenous fistula; pulmonary artery hypertension, with an estimated systolic blood pressure of 90 mmHg; significant right atrium and ventricle enlargement; and mitral and tricuspid valve insufficiency. The patient had not received any medical treatment prior to hospital admission. Routine blood tests, biochemical results, and arterial blood gas analysis were normal, with the exception of a PaO_2_ of 67 mmHg (Tables 1, 2, 3). The N‐terminal pro‐B‐type natriuretic peptide level—a guideline‐mandated biomarker of heart failure [6]—was high (Table 2).

**TABLE 1:** Preoperative routine blood tests.

Item	Result	Reference value
RBC	3.24 × 10^12^/L	3.5–5.5 × 10^12^/L
HCT	0.32	0.30–0.46
HGB	107 g/L	105–160 g/L
WBC	5.93 × 10^9^/L	4–10 × 10^9^/L
PLT	178 × 10^9^/L	80–300 × 10^9^/L
PLV	12.4 fl	6.0–11.5 fl
LY	1.2	0.8–4
NEU	4.29	2–7

Abbreviations: HCT, hematocrit; HGB, hemoglobin concentration; LY, lymphocytes; NEU, neutrophils; PLT, platelet count; PLV, platelet volume; RBC, red blood cells; WBC, white blood cells.

**TABLE 2:** Preoperative biochemical tests.

Item	Result	Reference value
K^+^	4.37 mmol/L	3.5–5.5 mmol/L
Na^+^	137.6 mmol/L	135–145 mmol/L
Cl^−^	101.3 mmol/L	96–108 mmol/L
Ca^2+^	2.24 mmol/L	2.0–2.8 mmol/L
TP	76.8 g/L	55–80 g/L
ALB	41.1 g/L	35–53 g/L
GLO	35.4 g/L	17.0–33.5 g/L
TBIL	7.9 μmol/L	3–25 μmol/L
GPT	67 U/L	0–64 U/L
AST	66 U/L	0–42 U/L
ALP	142 U/L	32–121 U/L
LDH	387 U/L	109–245 U/L
BUN	5.6 mmol/L	3.1–8.8 mmol/L
GLU	4.2 mmol/L	3.9–6.1 mmol/L
Cr	72.7 μmol/L	41.0–81.0 μmol/L
NT‐proBNP	628.9 pg/mL	< 300 pg/mL

Abbreviations: ALB, albumin; ALP, alkaline phosphatase; AST, glutamic oxaloacetic transaminase; BUN, blood urea nitrogen; Ca^2+^, calcium; Cl^−^, chloride; Cr, creatinine; GLO, globulin; GLU, glucose; GPT, glutamic–pyruvic transaminase; K^+^, potassium; LDH, lactate dehydrogenase; Na^+^, sodium; NT‐proBNP, N‐terminal pro‐B‐type natriuretic peptide; TBIL, total bilirubin; TP, total protein.

**TABLE 3:** Preoperative blood gas analysis.

Item	Result	Reference value
pH	7.40	7.35–7.45
PaO_2_	67.0 mmHg	80–100 mmHg
PaCO_2_	36.0 mmHg	35–45 mmHg
SaO_2_	93.1	95%–98%
${\mathrm{HCO}}_{3}^{-}$	21.9 mmol/L	22–27 mmol/L
${\mathrm{HCO}}_{3}^{-}$std	22.8 mmol/L	22–27 mmol/L
ABE	−1.8 mmol/L	−3 to 3 mmol/L
SBE	−2.1 mmol/L	−3 to 3 mmol/L
TCO_2_	51.6	50%–68%
T°C	37.0°C	36.5°C–37.5°C
FiO_2_	21.0	%

Abbreviations: ABE, actual base excess; FiO_2_, percentage of oxygen in inhaled gas; ${\mathrm{HCO}}_{3}^{-}$, bicarbonate ion; ${\mathrm{HCO}}_{3}^{-}$std, standard bicarbonate ion; PaCO_2_, partial pressure of carbon dioxide; PaO_2_, oxygen partial pressure; pH, potential of hydrogen; SaO_2_, oxygen saturation; SBE, standard base excess; T°C, temperature (degrees Celsius); TCO_2_, total carbon dioxide.

## 3 Methods

**FIGURE 3:**
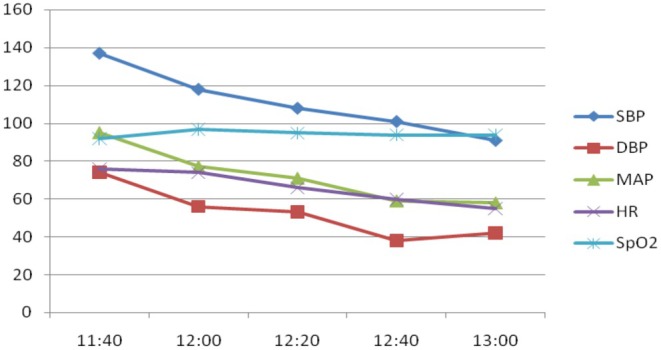
BP, MAP, HR, and SpO_2_ changes over time. DBP, diastolic blood pressure; HR, heart rate; MAP, mean arterial pressure; SBP, systolic blood pressure; SPO_2_, oxygen saturation.

**FIGURE 4:**
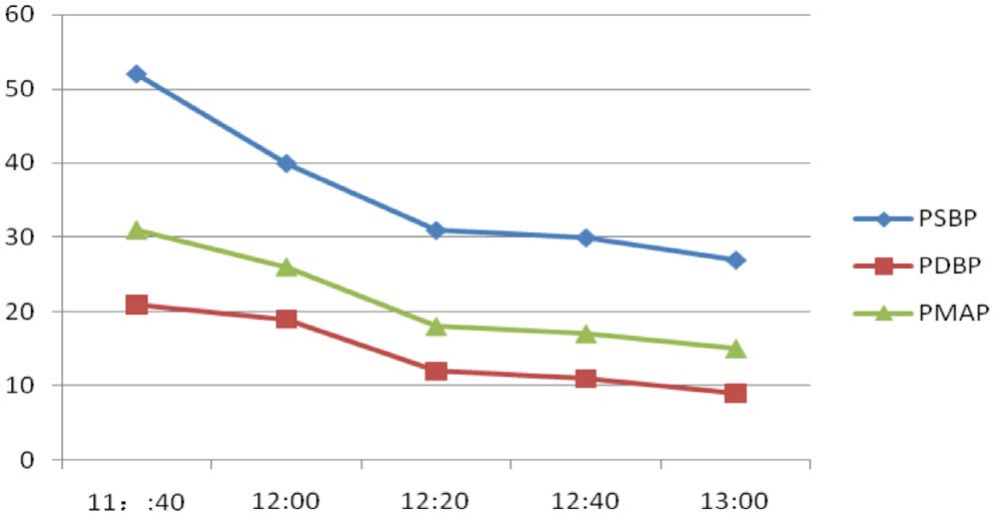
Pulmonary artery blood pressure changes over time. PDBP, diastolic blood pressure of the pulmonary artery; PMAP, mean arterial pressure of the pulmonary artery; PSBP, systolic blood pressure of the pulmonary artery.

Preoperatively, multidisciplinary discussion occurred between a cardiologist, obstetrician, intensive care doctor, cardiac surgeon, specialist doctor in extracorporeal membrane oxygenation, and echocardiographic doctor, among others. The patient was considered to be at high risk of right heart failure because of pulmonary hypertension. The application of VA‐ECMO was suggested. Upon entering the operating theater, the patient was placed supine on the operation table. All monitors, including heart rate (HR), noninvasive blood pressure (NIBP), and oxygen saturation (SpO_2_) measurement devices, were attached to the patient, and oxygen was initiated via a nasal cannula at 2 L/min. After local anesthesia with lidocaine, a 22‐G arterial catheter was inserted into the right radial artery for continuous BP monitoring. After local anesthesia with lidocaine, a Swan‐Ganz catheter (SGC) was inserted into the internal jugular vein under ultrasonographic guidance. The SGC floated into the pulmonary artery, guided by waveform variations from the pulmonary artery transducer. Her pulmonary arterial pressure (PAP) was 52/21 mmHg (mean, 31 mmHg). The anaesthesiologist decided to administer spinal anesthesia (SA) with ropivacaine, without VA‐ECMO assistance. The patient was placed in the left lateral decubitus position with her lumbar region curving backward. Following local anesthesia with lidocaine, a 25‐G spinal needle with a pen‐point was gently introduced between the third and fourth lumbar intervertebral space. Ropivacaine (12 mg) diluted to 3 mL with 10% glucose was injected into the subarachnoid space after free flowing, clear cerebrospinal fluid was observed at the end of the needle. Immediately after ropivacaine injection, the patient was placed in the supine position, with the operating table horizontal. The pain‐free region of anesthesia extended to the T7 vertebra. The patient was placed in the Trendelenburg position and adjusted until T4 sensory anesthesia was achieved. Obstetricians initiated the surgery after auscultation of the fetal sound. The neonate was successfully delivered 9 min after abdominal skin sterilization. A bag containing 3000 mL of normal saline wrapped in a sterile sheet was placed on the abdomen of the patient. NNI was immediately administered, with 1.3 mg (20 μg/kg) of nitroglycerin [7] diluted in 10 mL of 0.9% normal saline through a mask connected to a disposable inhaler, driven by oxygen at 6 L/min. Dexmedetomidine (65 μg, 1 μg/kg) diluted with saline to 10 mL was administered using a micropump for 10 min. BP and HR slightly decreased during the procedure, whereas SaO_2_ minimally changed (Figure 3). Similarly, PAP systolic pressure slowly decreased from 52 to 27 mmHg (Figure 4).

## 4 Results

The patient underwent successful cesarean delivery, with an estimated blood loss of 300 mL, urine output of 200 mL, and infusion of 500 mL Ringer's solution, without particular discomfort. After surgery, the patient was returned to the intensive care unit for postoperative recovery and monitoring. The patient was successfully discharged after 10 days.

## 5 Discussion

Pregnancy while suffering from pulmonary hypertension has been associated with a high mortality rate of 25% [7]. To the best of our knowledge, a gravida with severe heart disease and pulmonary arterial hypertension can experience complications during cesarean delivery. The returned blood volume suddenly increases after neonatal delivery, which can lead to acute right‐sided heart failure and even cardiac arrest. Mortality is higher if the increase in PAP is severe. Pulmonary hypertension is usually diagnosed using echocardiography [8]; nonetheless, a diagnosis made using a pulmonary catheter is considered the gold standard because of its higher precision and reliability [9]. Pulmonary hypertension is defined as a mean pulmonary artery pressure > 25 mmHg. Mild pulmonary hypertension is defined as a mean pulmonary artery pressure between 25 and 49 mmHg, whereas severe pulmonary hypertension is defined as a mean pulmonary artery pressure of ≥ 50 mmHg or a systolic pulmonary artery pressure of ≥ 70 mmHg, each based on the highest measured value during pregnancy [10, 11, 12]. The patient in the present case was diagnosed with severe pulmonary hypertension based on echocardiography showing a systolic pulmonary artery pressure of 90 mmHg. We measured the PAP using an SGC and obtained a result of 52/21 mmHg (mean 31 mmHg) before anesthesia. We considered VA‐ECMO to be unnecessary in this case; it would have been used if the mean PAP exceeded 50 mmHg or the systolic pressure exceeded 70 mmHg [13]. Venoarterial ECMO decreased the volume of the right heart, thereby decreasing the blood volume in the pulmonary artery and subsequently decreasing the PAP and right heart afterload. By this means, right heart function is protected [14].

Cesarean delivery is the predominant mode of delivery for pregnancies complicated by pulmonary hypertension. Expert opinion recommends planned cesarean delivery [15, 16, 17]. The anesthesia options are general anesthesia [18], regional anesthesia [19], spinal or epidural anesthesia, or combined spinal and epidural anesthesia. We selected SA with ropivacaine because it provides rapid and satisfactory anesthesia, good abdominal muscle relaxation, and stable hemodynamics [20, 21]. A 12‐mg dose of ropivacaine mixed with 10% glucose as a hyperbaric solution provided satisfactory anesthesia without inducing hypotension or necessitating vasopressor use in non‐cesarean delivery, as we observed clinically. Hypotension may be associated with aortocaval compression syndrome. Sensory blockage was faster in the left rather than the right lateral decubitus position on induction of spinal anesthesia [22]. Thus, we selected the left lateral decubitus position during the induction of SA.

Previously, a bag containing 3000 mL of normal saline packed in a sterile sheet was placed on the patients' abdomen after neonate parturition [23]. It was intended to slow the volume of blood returning to the right heart and prevent acute failure of the right heart.

Vasodilators are among the current therapeutic options for treating pulmonary hypertension. Inhalation of nitric oxide has also become a popular treatment; however, it may cause toxic and adverse reactions, and its administration requires expensive and complicated equipment [24]. Conversely, nebulized inhalation is easy to administer with a disposable inhaler driven by oxygen. Nebulized nitroglycerin is metabolized into nitric oxide, which is a potent smooth muscle relaxant in vascular endothelial cells, primarily in the lung, with minimal entry into systemic circulation [25]. To date, no study has indicated that nitroglycerin inhalation is toxic [26]. An intravenous infusion of nitroglycerin can be used to treat pulmonary hypertension; however, it decreases both the PAP and systemic blood pressure. NNI decreased the PAP and had little effect on the systemic blood pressure [27]. Therefore, we chose to use it in the patient in the current case. The PAP slowly declined after NNI administration, from 52/21 (31) mmHg at the beginning of the surgery to 27/9 (15) mmHg at the end. The slow decline in the systemic blood pressure may be related to dexmedetomidine administration via sympathetic nerve inhibition and a decreased HR. Patient anxiety about entering the operating room may have caused an increase in the BP at the beginning of the procedure. Dexmedetomidine provided adequate sedation during the surgery. We did not use a vasopressor to increase the BP because we considered that the returned blood volume would increase if the saline bag was moved. Inhaled nitroglycerin reduces pulmonary vascular resistance, which subsequently decreases the right ventricular afterload. It also improves oxygenation by selective vasodilation of the ventilated alveoli and relaxes the bronchial smooth muscles. However, we did not observe any change in the SpO_2_ during surgery. Moreover, blood loss and urine output cause fluid loss, which decreases the preload in the right heart. Accordingly, we transfused 500 mL of Ringer's liquid to balance the fluid loss.

## 6 Conclusion

In conclusion, we observed that NNI combined with dexmedetomidine during subarachnoid anesthesia decreased the PAP, prevented acute right heart failure, and increased safety during cesarean delivery in a parturient patient with pulmonary hypertension.

## Author Contributions

**Lian Xin:** conceptualization, writing – original draft. **Chunxiao Hu:** data curation, writing – review and editing. **Yiling Qian:** data curation, writing – review and editing. **Shunmei Lu:** data curation, writing – review and editing. **Jingjing Xu:** data curation, writing – review and editing. **Zhengfeng Gu:** project administration.

## Ethics Statement

This study was approved by the Ethics Committee of the Wuxi People's Hospital for Clinical New Technology and Scientific Study (KY24183).

## Consent

Written informed consent for publication was obtained from the patient discussed in this case report.

## Conflicts of Interest

The authors declare no conflicts of interest.

## Data Availability

Most of the data generated or analyzed in this case report is included in the article and its Supporting Information files. Additional data are available from the corresponding author upon request.
